# Microencapsulation via Spray-Drying of Geraniol-Loaded Emulsions Stabilized by Marine Exopolysaccharide for Enhanced Antimicrobial Activity

**DOI:** 10.3390/life13101958

**Published:** 2023-09-25

**Authors:** Ichrak Joulak, Samia Azabou, Emilie Dumas, Filomena Freitas, Hamadi Attia, Adem Gharsallaoui

**Affiliations:** 1 Laboratoire Analyse Valorisation et Sécurité des Aliments, Université de Sfax, ENIS, Sfax 3038, Tunisia; ichrak.joulak@gmail.com (I.J.); hamadi.attia@enis.tn (H.A.); 2 CNRS, LAGEPP UMR 5007, University of Lyon, Université Claude Bernard Lyon 1, 43 Bd 11 Novembre 1918, 69622 Villeurbanne, France; emilie.dumas@univ-lyon1.fr (E.D.); adem.gharsallaoui@univ-lyon1.fr (A.G.); 3 Associate Laboratory i4HB–Institute for Health and Bioeconomy, School of Science and Technology, NOVA University Lisbon, 2829-516 Caparica, Portugal; a4406@fct.unl.pt; 4 UCIBIO–Applied Molecular Biosciences Unit, Department of Chemistry, School of Science and Technology, NOVA University Lisbon, 2829-516 Caparica, Portugal

**Keywords:** exopolysaccharide, geraniol, oil/water emulsion, spray-drying, microencapsulation, antimicrobial activity

## Abstract

The current study investigates the formation of microencapsulated geraniol powder, with the exopolysaccharide EPS-K1B3 produced by *Halomonas caseinilytica* K1, as wall material, using spray-drying. Evaluation of the antimicrobial activity of the functional emulsions, prepared at either pH 5 or pH 7, was carried out against Gram-positive (*Listeria innocua* (ATCC 33090)) and Gram-negative (*Escherichia coli* (DSM682)) bacterial strains. Results showed prolonged antimicrobial efficacy until 30 days of incubation for geraniol microcapsules compared to wet geraniol emulsions, which could confirm the ability of the spray-drying process to protect encapsulated geraniol for a longer period. The highest antimicrobial efficacy of geraniol microcapsules was observed against *L. innocua* at pH 5. Therefore, the influence of pH on the functional property of geraniol microcapsules could be highlighted beside the targeted bacterial strain.

## 1. Introduction

Essential oils (EOs), which are mainly composed of aromatic and volatile compounds extracted from plants [1], are generally recognized as safe (GRAS) by the United States Food and Drug Administration [2]. Such compounds have been used since antiquity in various pharmaceutical, cosmetic, and food applications [3,4] for their strong antimicrobial, antiviral, anticancer, anti-inflammatory, and antioxidant activities [5,6,7,8]. EOs include natural bioactive compounds, such as asthymol, carvacrol, geraniol, eugenol, cinnamaldehyde, and citral [9]. Geraniol (3,7-dimethylocta-trans-2,6-dien-1-ol) is a commercially important acyclic monoterpene alcohol found in the EOs of several aromatic plants [10]. It is widely used by the flavour and fragrance industries due to its rose-like odour and taste (at 10 ppm), as well as its interesting biological activities that include antimicrobial, antioxidant, anti-inflammatory, antitumor, and insecticidal activities [11,12,13].

The antimicrobial activity of geraniol has been widely investigated and proven against various foodborne bacterial and fungal microbes. Therefore, geraniol has a high potential for commercial application as a growth-inhibiting agent [14,15]. In fact, Lira et al. [16] have recently listed in their review 78 microbial species, covering a wide spectrum of fungal and bacterial genera that are sensitive to geraniol. In addition, among 66 essential oils/compounds tested, geraniol exhibited significant growth-inhibiting activity against both human and animal pathogens, *Salmonella typhimurium* and *Escherichia coli* [17]. Owing to its hydrophobicity, geraniol can disrupt the lipids of the bacterial cell membrane and mitochondria, which render cell structures more permeable; extensive leakage of critical molecules and ions from bacterial cells will lead to death [18]. In addition, geraniol is claimed as GRAS because of its high efficiency, safety, and non-toxicity. Thus, geraniol could be a potential EO to apply in food products as a natural antimicrobial agent [14].

However, mainly due to their volatile nature and low chemical stability, EOs are often degraded when directly applied to food systems. Therefore, several studies have focused on developing delivery systems to encapsulate EOs aiming to protect them and to enhance their stability [19]. Delivery systems can be formed by emulsification, spray-drying, freeze-drying, in situ polymerization, coacervation, extrusion, coaxial electrospray system, fluidized-bed-coating, and supercritical fluid technology [20]. Among these techniques, spray-drying is the most commonly used drying technology in the food industry due to its low cost and available equipment. Depending on the encapsulated material and the characteristics desired in the final product, wall materials can be selected from a wide variety of natural and synthetic polymers or monomers. This process makes it possible to produce powdered microcapsules from liquid in a single simple and scalable operation. It is possible to prepare mixtures of active molecules and biopolymers, and the wet suspensions/emulsions will then be transformed into powders by evaporating the majority of the water when the formed droplets are in contact with hot air [21].

Emulsification is one of the most important processes in the food and pharmaceutical industries, allowing the encapsulation of a hydrophilic or lipophilic active molecule inside a dispersed phase, thus ensuring its physicochemical stability and protection against environmental stress and degradation and allowing its controlled delivery [22]. Nevertheless, emulsions are not thermodynamically stable and are therefore prone to destabilization caused by gravitational separation, flocculation, and/or coalescence during long term-storage [23]. Stable emulsions can be obtained by the addition of stabilizing agents, such as surface-active emulsifiers, which adsorb at the interface between the two phases, decrease the interfacial tension, and dissipate the excess surface free energy at this newly formed interface [24]. They can also stabilize emulsions by forming a thin and protective layer around the dispersed droplets, which prevents the phenomenon of coalescence [23].

Various synthetic molecules commonly used as emulsifiers can, directly or indirectly, cause toxicity and environmental problems [25]. In line with the increasing tendency for using natural food ingredients, synthetic emulsifiers are gradually being replaced by naturally occurring compounds, including biopolymers. Among these, microbial exopolysaccharides (EPS) are valuable alternative stabilizer agents for emulsions that act by increasing the continuous aqueous phase viscosity, thus providing steric effects or forming a gel [26,27,28]. EPS are high molecular weight polymers that are produced by yeasts, fungi, algae, archaea, or bacteria [29]. They can be found either as capsular materials attached to the microbial cell or as a slime dispersed in the cell’s surrounding environment. Due to their wide chemical and structural diversity, EPS from halophilic bacteria have shown distinctive functional properties that make them promising candidates in various fields. Currently, there is a great deal of interest on their application in the food sector as viscosifying agents, stabilizers, emulsifiers, gelling agents, or water-binding agents [30].

In the present work, oil/water (O/W) emulsions were prepared to encapsulate geraniol using EPS-K1B3, produced by the halophilic strain *Halomonas caseinilytica* K1, as a stabilizing agent. The formed emulsions were spray-dried to obtain microcapsules, whose antimicrobial activity was evaluated against both Gram-negative and Gram-positive bacteria. Thus, the objectives of this study were (i) to evaluate the effectiveness of EPS-K1B3 to microencapsulate geraniol, (ii) to characterize the physicochemical properties of geraniol microcapsules, and (iii) to evaluate their antimicrobial activity before and after microencapsulation.

## 2. Materials and Methods

### 2.1. Materials

EPS-K1B3 has been previously produced by the fed-batch cultivation of Halomonas caseinilytica K1 (JCM 32859). Cheese whey was supplied by Lactogal Produtos Alimentares S.A. (Porto, Portugal). Sodium caseinate was acquired from Fisher Scientific (Loughborough, UK). Maltodextrins DE 19 (dextrose equivalent value of 19) was obtained from Roquette-freres SA, (Lestrem, France). Geraniol (trans-3,7-Dimethyl-2,6-octadien-1-ol), imidazole (C3H4N2), acetic acid, sodium hydroxide (NaOH), hydrochloric acid (HCl), and ethanol were purchased from Sigma-Aldrich Chimie (St Quentin Fallavier, France). Distilled water was used for the preparation of all solutions. Bacterial strains, namely, *L. innocua* (ATCC 33090) and *E. coli* (DSM682) were used as target strains for the antimicrobial assessment. Pure bacterial cultures were revived from −80 °C in tryptone soy broth (TSB, Biokar Diagnistics, Allonne, France).

### 2.2. Production, Extraction and Characterization of EPS-K1B3

EPS-K1B3 was produced by the fed-batch cultivation of *Halomonas caseinilytica* K1 (JCM 32859) in a semi-complex medium (SCM) [31] and supplemented with cheese whey (8%, *w*/*v*) (supplied by Lactogal Produtos Alimentares S.A., Porto, Portugal) as the sole carbon source. Production was conducted in 2 L bioreactors (BioStat B-plus, Sartorius, Germany) under controlled conditions of pH (7.0 ± 0.05), temperature (30 ± 0.1 °C), and dissolved oxygen (DO) concentration (20%). The DO was controlled by the automatic variation of the substrate-feeding rate, which was gradually increased from 5 mL/h (initiated at 6 h-cultivation) to 25 mL/h (at 33–48 h). An EPS concentration of 11.67 ± 0.70 g/L was obtained at the end of the 48-h cultivation run. The secreted EPS was recovered from the cell-free supernatant by dialysis with a 10,000 MWCO membrane (SnakeSkin^TM^ Pleated Dialysis Tubing, Thermo Scientific, Waltham, MA, USA) against deionized water. The obtained biopolymer was lyophilized and characterized in terms of its composition in sugars [32], acyl groups [32], phosphate and sulphate [33], molecular mass distribution [32], and total carbohydrates [31], as well as its contents in moisture, inorganic salts, and total protein. The moisture content of the purified EPS was determined by estimating its weight loss after oven drying at 105 °C until a constant weight was obtained. Afterward, the dried sample was placed at 550 °C for 2 h, and the inorganic salt content was determined gravimetrically by weighing the resulting ashes. The Bradford method [34] was carried out to determine the protein content.

### 2.3. Preparation of EPS Stabilized O/W Emulsions

O/W emulsions were prepared under sterile conditions by initially dissolving lyophilized EPS-K1B3 (0.5%, *w*/*v*) in imidazole/acetate buffer (5 mM) at pH 5.0 and at pH 7.0. The mixtures were stirred overnight with a magnetic stirrer until complete dissolution. Then, the pH of EPS solutions was adjusted by adding HCl (0.1 M) or NaOH (0.1 M). Afterwards, geraniol (trans-3,7-Dimethyl-2,6-octadien-1-ol) (Sigma-Aldrich Chimie, St Quentin Fallavier, France) was added as the antimicrobial agent at a final concentration of 10% (*w*/*w*). To form emulsions, geraniol was slowly incorporated into EPS-K1B3 solutions by stirring at 20,000 rpm for 10 min, using an Ultra Turrax T-18 basic (IKA, Staufen, Germany). Blank emulsions containing sunflower oil were also prepared in the same conditions. All the emulsions were readjusted to the suitable pH before measurements.

### 2.4. Spray-Drying Process

Stock maltodextrins (DE 19, dextrose equivalent value of 19; Roquette-freres SA, Lestrem, France) solutions (40%, *w*/*w*) were added to the wet O/W emulsions (pH 5.0 and 7.0) and kept under continuous agitation for at least 2 h. To form the microcapsules, the obtained mixtures (final composition (*w*/*w*) of 20% maltodextrins DE 19; 0.25% EPS and 5% oil) were stirred and then spray-dried using a laboratory-scale machine (Mini spray-dryer B-290, Büchi, Switzerland) equipped with a nozzle atomizer (0.5 mm diameter). The emulsions were fed into the spray-dryer at a flow rate of 0.5 L/h, at 25 °C. The inlet and outlet temperatures were 180 ± 2 °C and 81 ± 9 °C, respectively. The dried powders were collected and stored at 4 °C for further analysis.

### 2.5. Zeta Potential Measurement

The zeta potential (ζ-potential) of EPS-K1B3 as a function of pH, as well as the ζ-potential of the O/W emulsions before spray-drying (prepared as mentioned in the following section) were determined using a Zetasizer NanoZS90 instrument (Malvern Instruments, Malvern, UK). The mean ζ-potential values (±standard deviation) were obtained from the instrument.

### 2.6. Interfacial Tension Measurement

An automatic drop tracker tensiometer (Teclis Scientific, Civrieux-d’Azergues, France) was used to measure the interfacial tension at the O/W interface, at 25 °C. The aqueous phase (W) containing the EPS (0.01%, *w*/*v*) was prepared in imidazole/acetate buffer (5 mM) and adjusted at either pH 5.0 or pH 7.0, and the oily phase (O) was geraniol oil. Sodium caseinate (Cas) (Fisher Scientific, Loughborough, UK) prepared in the same conditions was used as a reference.

### 2.7. Particle Size Measurement

Particle size of the wet emulsions and the spray-dried microcapsules was assessed using a laser diffraction instrument (Mastersizer 3000, Malvern Instruments, Malvern, UK) with a value of 1.5 for relative refractive index (droplet to solvent), 0.1 absorption, and refraction indexes of 1.33 and 1.36 for water and ethanol, respectively. For droplet size measurement, the wet emulsions were diluted in imidazole/acetate buffer (5 mM) and adjusted to the suitable pH to avoid multiple scattering effects. The droplet size was calculated by volume mean particle diameter (D [4,3]), using the following Equation (1):

D [4,3] = (∑ n_i_ Di ^4^)/(∑ n_i_ D_i_ ^3^) (1)

where Di: diameter of class “i” and n_i_: number of particles of class “i” [35].

For measuring the particle size distribution of the spray-dried microcapsules, the powders were slowly added into the measurement chamber containing absolute ethanol. The emulsions and spray-dried powders were continuously stirred to ensure good homogenization of the sample.

### 2.8. Evaluation of the Antimicrobial Activity of the Wet Emulsions and Spray-Dried Microcapsules

Two bacterial strains, the Gram-positive *L. innocua* (ATCC 33090) and the Gram-negative *E. coli* (DSM682), were used as target strains for the antimicrobial assessment. *L. innocua* and *E. coli* were reactivated and sub-cultured in TSB at 10% (*v*/*v*) for 7 h and then pre-cultured in TSB at 10% (*v*/*v*) for 17 h at 37 °C. After that, 1 mL of the pre-culture was transferred into 9 mL TSB and incubated for 5 h at 37 °C. Bacterial cells were then diluted in tryptone salt (TS) broth to a final concentration of 1 × 10^6^ CFU/mL, incorporated at 5% (*v*/*v*) in melted tryptone soy agar (TSA) and cooled in Petri dishes. To evaluate the antimicrobial activity of the wet emulsions, wells (10 mm in diameter) were made by a sterilized Pasteur pipette onto the TSA Petri plates previously inoculated with *L. innocua* or *E. coli*, and 50 µL of the emulsion sample was placed in the well. The antimicrobial activity of the spray-dried microcapsules was assessed by placing a sterilized filter paper with a hole (15 mm in diameter) on the TSA Petri plates inoculated with *L. innocua* or *E. coli*, and adding weighted amounts (30 µg) of the microcapsules in the hole. After spreading the powder, the paper was gently removed. The plates were then incubated for 2 h at 4 °C and then incubated for 24 h at 37 °C. Afterwards, inhibition zones were calculated by subtracting the diameter of the total inhibition zone from the diameter of the well and the hole [26]. Each experiment was repeated three times. Wet emulsions and spray-dried microcapsules containing sunflower oil were used as blanks.

### 2.9. Kinetics of Inhibition Zones Diameters of Wet and Spray-Dried Emulsions

Growth inhibition zones diameters around emulsions, which were formed at both pH 5 and pH 7, were measured during 30 days of incubation, as reported by Ben Amara et al. [36].

### 2.10. Statistical Analysis

All experiments were performed at least three times. The results presented were expressed in means ± standard deviations that were calculated from these replicate measurements. Statistical study was performed using the software IBM SPSS 1 V. 21. Differences between samples were considered statistically significant when *p* < 0.05 at confident level of 95%.

## 3. Results and Discussion

### 3.1. Physicochemical Properties of EPS-K1B3

#### 3.1.1. Chemical Characterization

The EPS-K1B3 sample used in this study as emulsion stabilizer comprised a carbohydrate content of 69.36 ± 1.20%, with lower contents of protein (9.47 ± 0.12%) and inorganic salts (11.78 ± 0.73%), and a moisture of 7.20 ± 1.58% (Table 1). The polysaccharide had a molecular weight of 2.25 × 10^5^ Da and polydispersity index of 2.10, and was composed of rhamnose (32 mol%), galactose (24 mol%), glucose (17 mol%), mannose (11 mol%), and ribose (8 mol%), with minor contents of glucuronic acid (3 mol%) and arabinose (5 mol%).

Moreover, it contains phosphate (5.41 ± 0.09%), sulphate (1.73 ± 0.06%), and acyl groups (0.99 ± 0.08%), along with glucuronic acid residues, which influence the net charge of the EPS sample.

#### 3.1.2. Zeta Potential

**Figure 1:**
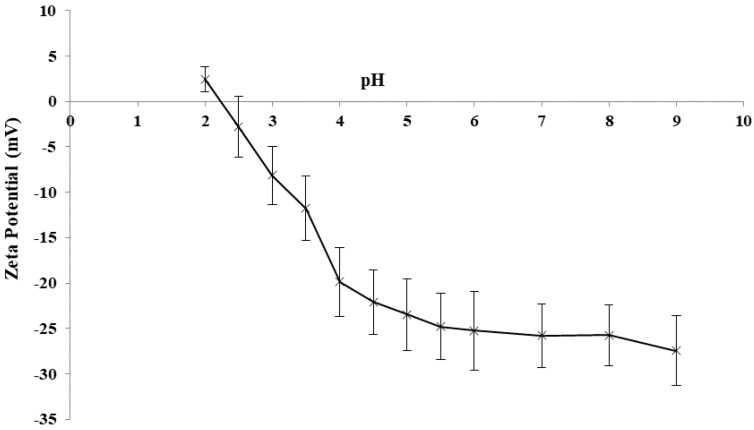
Zeta potential of EPS-K1B3 (1 g/L) as a function of pH (from 2 to 9). Error bars = least significant differences (*p* < 0.05, n = 3).

Measuring the zeta potential of EPS-K1B3 at pH values ranging from 2 to 9 (Figure 1) showed that this EPS was negatively charged at the different pH values, except at pH 2. The anionic property of EPS-K1B3 is associated with the presence of glucuronic acid, phosphate, and sulphate residues containing negatively charged functional groups.

During the last decades, new EPS producers have been incessantly investigated in order to recover original EPS with novel composition, and therefore possess attractive characteristics to be exploited in food, cosmetic, and pharmaceutical industries as bio-flocculants, bio-absorbents, and drug delivery [30,31]. Taking into account that EPS-K1B3 is a rhamnose-rich polysaccharide showing anionic property, these structural features can promote apolar self-association and lower surface and interfacial tension and therefore impart stability to O/W emulsions and could be used as an ideal polymer to prepare nanocarriers.

#### 3.1.3. Interfacial Tension

**Figure 2:**
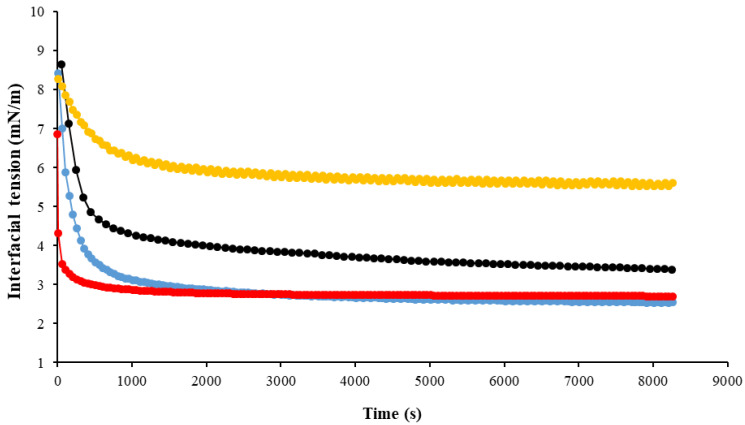
Time-dependent changes of the interfacial tension of EPS-K1B3 (0.01%) and Cas samples (0.01%) at pH 5 and pH 7. EPS-K1B3 at pH 5 (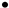), EPS-K1B3 at pH 7 (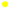), Cas at pH 5 (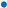), and Cas at pH 7 (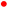).

In order to evaluate its emulsifying properties, the interfacial tension of EPS-K1B3 (0.01%) at the O/W interface was determined at both pH 5 and pH 7, and compared to that of sodium caseinate (Cas) as control. The interfacial tension of EPS and Cas displayed a sharp decrease within the first 1000 s, and the adsorption equilibrium was reached at the end of the measurement time (Figure 2). This indicated the rapid adsorption of the biopolymer’s macromolecules to the O/W interface. Such phenomenon is a typical characteristic of proteins or protein/polysaccharide complexes [37]. However, scarce studies have reported the interfacial properties of native EPS only [38].

Different adsorption behaviours as a function of pH can also be observed. In fact, the interfacial tension between geraniol and EPS-K1B3 aqueous solution at pH 7 decreased from 8.27 to 5.60 mN/m, while it decreased from 8.65 to 3.38 mN/m at pH 5 throughout the measurement time. Therefore, it can be noted that EPS-K1B3 had higher adsorption affinity to the O/W interface at pH 5 compared to that at pH 7. Otherwise, EPS-K1B3 displayed better surface activity at an acidic pH than at a neutral pH. Opposite results were observed by Abid et al. [38] that reported that the interfacial tension of another type of EPS (EPS-GM) was not affected by the pH. This may be due to the different chemical composition of EPS-GM sample. According to these results, EPS-K1B3 could be used as natural emulsifier and stabilizer in both acidic and neutral emulsions.

### 3.2. Physicochemical Properties of EPS-K1B3 Stabilized Emulsions

#### 3.2.1. Droplet Size Distribution and Zeta Potential of Wet Emulsions

The droplet size and zeta potential of emulsions containing 0.5% EPS and 10% oil are presented in Table 2.

It can be noticed that there is no significant (*p* > 0.05) difference between the droplet size of the geraniol emulsion formed at pH 5 and that of sunflower emulsion formed at the same pH. Moreover, the droplet size of the geraniol emulsion formed at pH 7 (24.95 ± 2.45 μm) was significantly (*p* < 0.05) higher than that of the geraniol emulsion formed at pH 5 (7.25 ± 1.80 μm). As reported by Horozov et al. [39], larger droplets may result in less stable emulsions, which could be mainly caused by the aggregation between emulsion droplets. This phenomenon results from the attractive forces between the droplets and lead to the formation of flocs (flocculation) and/or larger droplets (coalescence). As seen in Table 2, sunflower emulsions formed at both pH 5 and pH 7 were characterized by high zeta potential values (ranging from −36.80 ± 3.52 mV to −43.30 ± 3.41 mV) and their droplet size was small (ranging from 4.66 ± 0.12 µm to 3.64 ± 0.19 µm). It can be expected that relatively high surface charges increased the electrostatic repulsion and avoided the aggregation between emulsion droplets and, thus, improved the emulsion’s stability. However, Table 2 shows that, although the negative surface charges of the geraniol emulsion at pH 7 were important (−33.65 ± 4.37 mV), their particle size was relatively large (24.95 ± 2.45 µm), indicating less stability of the emulsion. Thus, it can be noted that the emulsion’s stability was not only related to the surface charges, but also to the interfacial properties of the EPS. In fact, as mentioned above, EPS-K1B3 displayed better surface activity at pH 5 than at pH 7 (Figure 2).

#### 3.2.2. Particle Size Distribution of Emulsions before and after Spray-Drying

The particle size distribution of emulsions containing 0.25% EPS, 5% oil, and 20% maltodextrins DE 19, prepared at pH 5 and pH 7, was evaluated before and after spray-drying (Table 3).

**Figure 3:**
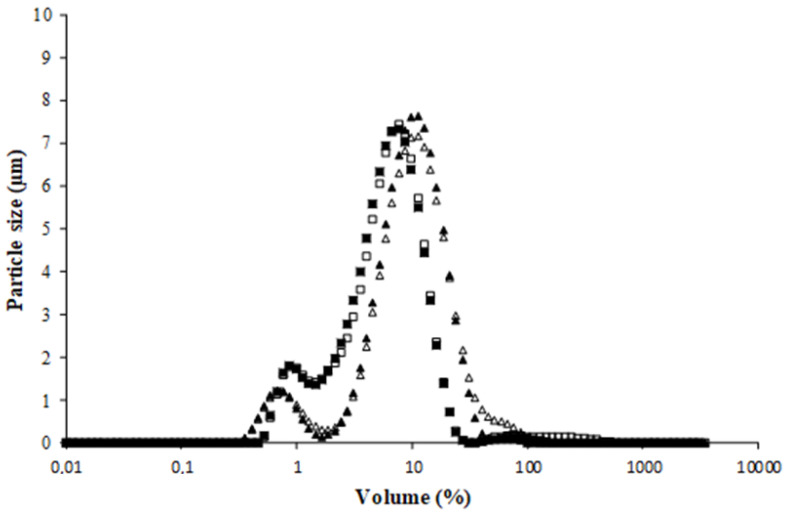
Droplet size distribution of emulsions after spray-drying. Sunflower emulsion at pH 5 (Δ), sunflower emulsion at pH 7 (▲), geraniol emulsion at pH 5 (☐), and geraniol emulsion at pH 7 (■).

The droplet size of the sunflower emulsions which were formed at pH 5 and pH 7 increased after spray-drying (from 4.81 ± 0.90 to 11.49 ± 0.40 µm at pH 5; from 3.76 ± 1.20 µm to 11.75 ± 2.10 µm at pH 7). This could be mainly caused by flocculation and/or coalescence induced during the spray-drying process and particularly during the heating step. On the other hand, the droplet size of the geraniol emulsions formed at pH 5 and pH 7 decreased after spray-drying (from 20.40 ± 2.84 to 11.73 ± 3.75 at pH 5; from 25.80 ± 3.44 to 10.01 ± 2.86 µm). This could be explained by the dissociation of aggregated droplets during the shearing inside the atomization nozzle, the collapse of surface hairy layers, and the shrinkage of overall structures [40]. It can be noted that there is no significant difference (*p* > 0.05) between the final particle size of emulsions after spray-drying, since all the formed emulsions had almost the same particle size distribution (Figure 3).

### 3.3. Antimicrobial Properties of EPS-K1B3 Stabilized Emulsions

**Figure 4:**
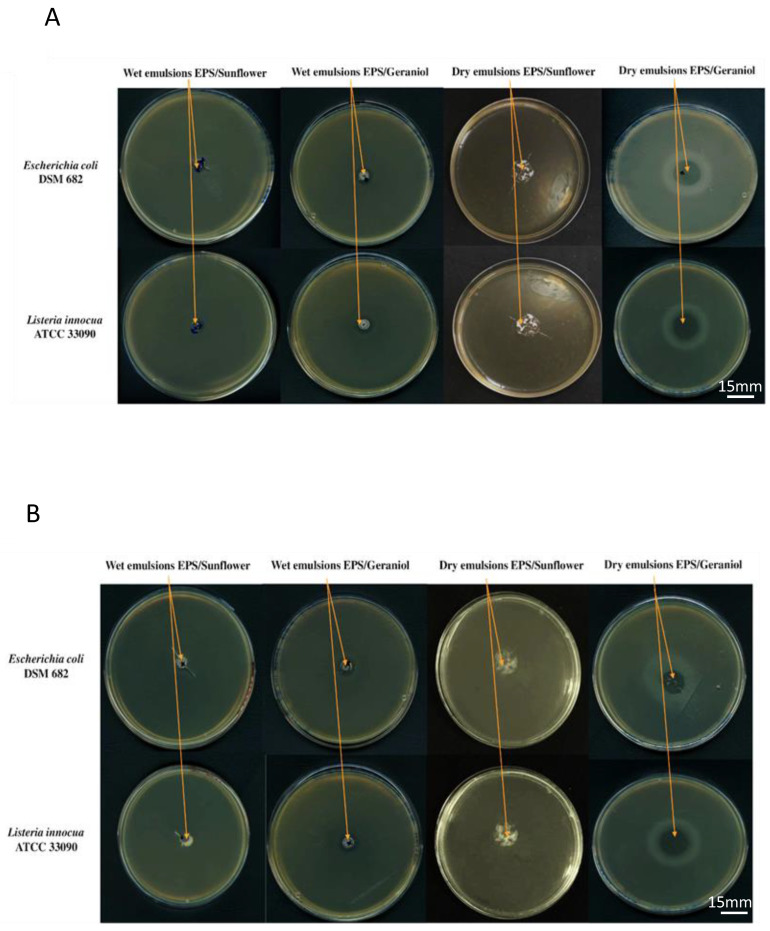
Antimicrobial properties of EPS-K1B3 stabilized emulsions: Petri dishes after 24 h containing TSA medium and *E. coli* or *L. innocua* with wet or spray-dried emulsions at pH 5 (**A**) and pH 7 (**B**). Antimicrobial activity was evidenced by a growth inhibition zone measured after 24 h of incubation at 37 °C.

The antimicrobial activity of the wet emulsions prepared at pH 5 and pH 7, as well as the spray-dried microcapsules, was evaluated against the Gram-positive bacterium, *Listeria innocua* ATCC 33090, and the Gram-negative bacterium, *Escherichia coli* DSM682, using the agar well diffusion method. The wet emulsions were added into the wells and microcapsules were deposed on the surface of TSA medium containing bacteria. Photographs of Petri dishes after 24 h incubation, containing wet and dry emulsions prepared at pH 5 and pH 7, are shown in Figure 4A and Figure 4B, respectively.

Wet and dry emulsions containing geraniol were effective against the two target bacteria as evidenced by the growth inhibition zones. However, there were no inhibition zones around wet and dry emulsions containing sunflower oil (blank) (Figure 4). Therefore, it can be concluded that EPS-K1B3 alone does not possess any antimicrobial activity against the tested strains.

Considering the inhibition zones diameters around wet emulsions, it can be concluded that geraniol emulsions formed at pH 5 and pH 7 had the same antimicrobial activities (inhibition zone diameter was 2 mm) against *L. innocua* and *E. coli* (Figure 4), indicating that the changes of pH does not affect their antimicrobial activity. On the other hand, after using the same amount of dry emulsions prepared at pH 5 and pH 7, the inhibition zones around the powders after 24 h of incubation were less significant than those around wet emulsions (Figure 4), regardless of the tested microorganism. This is probably due to the diffusion time of the encapsulated geraniol in the agar. Furthermore, geraniol microcapsules prepared at pH 5 had a larger inhibition zone diameter (1 mm) than those formed at pH 7 (0.5 mm) against *E. coli.* Moreover, geraniol microcapsules prepared at pH 5 were more efficient against *L. innocua* than those obtained at pH 7 (Figure 4). It can be noted that the effectiveness of geraniol microcapsules was more significant at pH 5 than at pH 7, regardless of the tested bacterial strain.

**Figure 5:**
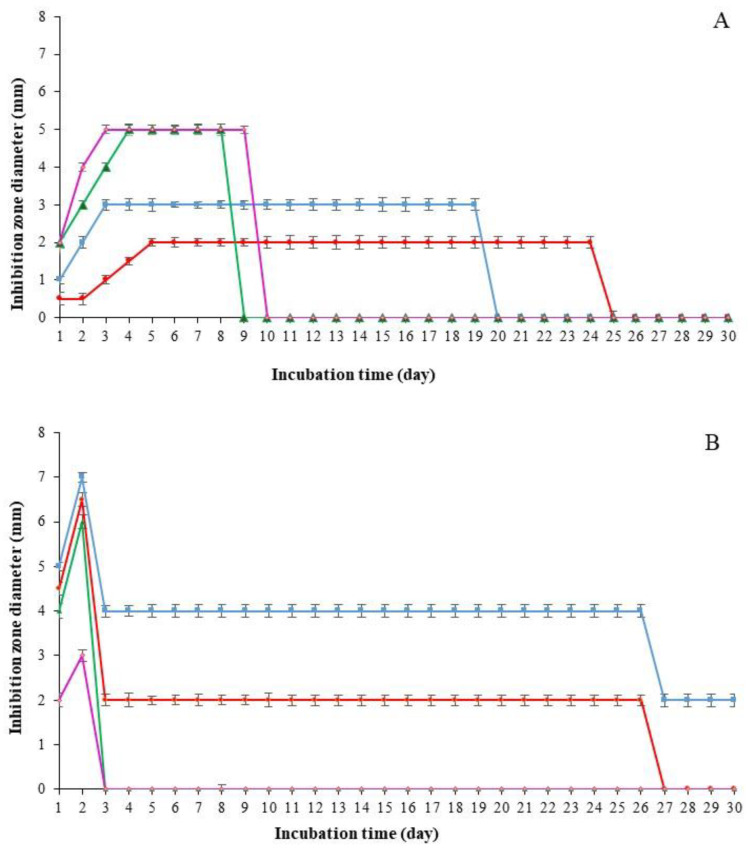
Kinetics of the antimicrobial activity (growth inhibition zones) during the storage of wet and dry emulsions made at different pH values against *E. coli* (**A**) and *L. innocua* (**B**). Dry emulsions at pH 5 (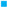), dry emulsions at pH 7 (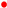), wet emulsions at pH 5 (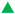), and wet emulsions at pH 7 (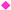). Error bars = least significant differences (*p* < 0.05, n = 3).

Kinetics of inhibition zones diameters around wet and dry emulsions were evaluated during 30 days of incubation. Figure 5A,B show the kinetic of inhibition zones diameters against *E. coli* and against *L. innocua*, respectively. As shown in Figure 5, the antimicrobial activity of the wet and dry emulsions against the two target strains displayed different profiles over time.

The geraniol microcapsules prepared at pH 5 were more efficient against *E. coli* than those obtained at pH 7. Nevertheless, the inhibition zone around geraniol microcapsules at pH 7 persisted longer (24 days) than those at pH 5 (20 days) (Figure 5A). Moreover, the geraniol microcapsules formed at pH 5 were more efficient against *E. coli* than against *L. innocua* (Figure 5). However, this activity against *L. innocua* was maintained during the 30 days of incubation for the samples prepared at pH 5, while it persisted only for 26 days for those prepared at pH 7 (Figure 5B). It could also be noticed that geraniol microcapsules were efficient at pH 7 against *E. coli* and at pH 5 against *L. innocua*. This indicated that the effectiveness of geraniol microcapsules depended not only on the pH value during emulsion preparation, but also on the tested bacterial strains. Noticeably, Figure 5 also shows that the inhibition zones for the wet emulsions disappeared in shorter incubation periods than that of the spray-dried emulsions. As mentioned above, the diffusion of the encapsulated geraniol in agar and its release took longer for powders than for liquids. Interestingly, these results demonstrated that the spray-drying process was able to protect the encapsulated geraniol and to maintain its antimicrobial activity for a long period. Therefore, the formed geraniol microcapsules could be a good food preservative, even when used at non-inhibitory doses, as they slowdown microbial growth. It can also be noted that inhibition zones against *E. coli* around wet emulsions persisted longer (9 days) than those of wet emulsions against *L. innocua* (2 days), indicating that liquid emulsions were more efficient against *E. coli* than against *L. innocua* at both pH 5 and pH 7.

The application of EOs as an alternative to control pathogen microorganisms is limited mainly due to their high volatility; therefore, they should be protected by a physical barrier which slows down the evaporation rate and ensures its controlled release. In this sense, microencapsulation is a powerful way to control the release of antimicrobial agents to achieve better efficiency and a longer-lasting effect [21]. In the same vein, Chen and Vilgoen [15] have recently deeply reviewed how encapsulating could enhance both stability and effectiveness of geraniol and therefore increase its antimicrobial potency. Nevertheless, prior to the application of the studied microcapsules, the EPS-producer *Halomonas caseinilytica* K1 should be submitted in the future to an assessment of its potential pathogenicity in order to guarantee the suitability of the culture for use at industrial/commercial level.

## 4. Conclusions

Results from the present study showed that the stability of emulsions was affected by the pH value during their preparation. In fact, EPS-K1B3 had better interfacial properties at pH 5 than at pH 7, which makes its emulsifying and stabilizing properties more efficient at pH 5. Microbiological tests demonstrated that the antimicrobial activity of the emulsions depended on both the pH used for their preparation and on the target microorganism. Moreover, spray-dried microcapsules were more efficient than wet emulsions over 30 days of incubation. Therefore, spray-drying is a suitable encapsulation technique for preserving the functional characteristics of geraniol and ensuring its controlled release. These formulated geraniol microcapsules could be used in food and cosmetic industries as natural substances for improving flavour and aroma retention in a cosmetic or a food product, as well as for extending shelf-life.

## Figures and Tables

**Table 1:** Chemical characterization of EPS-K1B3 sample.

Parameter	Value (%)
Moisture	7.20 ± 1.58
Total carbohydrate	69.36 ± 1.20
Total protein	9.47 ± 0.12
Inorganic salts	11.78 ± 0.73

Data are presented as the mean ± standard deviation (n = 3).

**Table 2:** Physicochemical properties of geraniol and sunflower wet emulsions (containing 0.5% EPS and 10% oil) formed at pH 5 and pH 7.

	Geraniol Emulsions		Sunflower Emulsions	
	pH 5	pH 7	pH 5	pH 7
D [4,3] µm	7.25 ^a^ ± 1.80	24.95 ^b^ ± 2.45	4.66 ^a,c^ ± 0.12	3.64 ^c^ ± 0.19
Zeta potential (mv)	−25.90 ^a^ ± 3.59	−33.65 ^b^ ± 4.37	−36.80 ^b,c^ ± 3.52	−43.30 ^c^ ± 3.41

Data are presented as the mean ± standard deviation (n = 3) and values with different letters in the same line are significantly different according to Duncan’s multiple range test (*p* < 0.05).

**Table 3:** Particle size distribution of geraniol and sunflower emulsions (containing 0.25% EPS, 5% oil, and 20% maltodextrins DE 19) formed at pH 5 and pH 7, before and after spray-drying.

		Geraniol Emulsions		Sunflower Emulsions	
		pH 5	pH 7	pH 5	pH 7
D [4,3] µm	Before spray-drying	20.40 ^a^ ± 2.84	25.80 ^b^ ± 3.44	4.81 ^c^ ± 0.90	3.76 ^c^ ± 1.20
	After spray-drying	11.73 ^d^ ± 3.75	10.01 ^d^ ± 2.86	11.49 ^d^ ± 0.40	11.75 ^d^ ± 2.10

Data are presented as mean ± standard deviation (n = 3). The letters represent the statistical difference between the droplet size of the emulsions formed at pH 5 and 7 before and after the spray-drying process (same letter: *p* > 0.05).

## Data Availability

Data will be made available on request.
